# Strategies, Actions, and Outcomes of Pilot State Programs in Public Health Genomics, 2003–2008

**DOI:** 10.5888/pcd11.130267

**Published:** 2014-06-12

**Authors:** Jeanette St. Pierre, Janice Bach, Debra Duquette, Kristen Oehlke, Robert Nystrom, Kerry Silvey, Amy Zlot, Rebecca Giles, Jenny Johnson, H. Mack Anders, Marta Gwinn, Scott Bowen, Muin J. Khoury

**Affiliations:** Author Affiliations: Jeanette St. Pierre, Muin J. Khoury, Centers for Disease Control and Prevention, Atlanta, Georgia; Janice Bach, Debra Duquette, Michigan Department of Community Health, Lansing, Michigan; Kristen Oehlke, Minnesota Department of Health, St Paul, Minnesota; Robert Nystrom, Kerry Silvey, Amy Zlot, Oregon Health Authority, Portland, Oregon; Rebecca Giles, Jenny Johnson, Utah Department of Health, Salt Lake City, Utah; H. Mack Anders, Marta Gwinn, McKing Consulting Corporation, Atlanta, Georgia.

## Abstract

State health departments in Michigan, Minnesota, Oregon, and Utah explored the use of genomic information, including family health history, in chronic disease prevention programs. To support these explorations, the Office of Public Health Genomics at the Centers for Disease Control and Prevention provided cooperative agreement funds from 2003 through 2008. The 4 states’ chronic disease programs identified advocates, formed partnerships, and assessed public data; they integrated genomics into existing state plans for genetics and chronic disease prevention; they developed projects focused on prevention of asthma, cancer, cardiovascular disease, diabetes, and other chronic conditions; and they created educational curricula and materials for health workers, policymakers, and the public. Each state’s program was different because of the need to adapt to existing culture, infrastructure, and resources, yet all were able to enhance their chronic disease prevention programs with the use of family health history, a low-tech “genomic tool.” Additional states are drawing on the experience of these 4 states to develop their own approaches.

## Background

The Human Genome Project concluded in 2003, attracting attention to genomic research as a source for health information and applications (1). State public health departments already had well-established programs to screen newborns for rare genetic disorders, provide clinical genetic services to underserved populations, and conduct surveillance for birth defects and developmental disabilities. State programs for chronic disease prevention and health promotion were focused on monitoring health problems, promoting healthful behaviors, and assuring access to preventive services. Using genomic information in these programs was largely uncharted territory (2).

To explore the potential use of genomic information in chronic disease prevention, the Centers for Disease Control and Prevention supported pilot programs in 4 states from 2003 through 2008. We describe the strategies, successes, challenges, and consequences of these programs, which offer potential models for using genomic information — particularly family health history — for public health action.

## Methods

### Cooperative agreements

The Chronic Disease Directors’ Summit in 2002 called on CDC to help states respond to knowledge and applications emerging from the Human Genome Project (3). In 2003, CDC announced the availability of funding for public health genomics through a cooperative agreement with CDC’s National Center for Chronic Disease Prevention and Health Promotion (4). The same funding announcement also included 6 other components of chronic disease prevention: tobacco; nutrition, physical activity, and obesity; Well-Integrated Screening and Evaluation for Women Across the Nation (WISEWOMAN); oral health; arthritis; and the Behavioral Risk Factor Surveillance System. The intent of this bundled announcement was to encourage collaboration among programs on common objectives and cross-cutting risk factors. State health departments and other eligible organizations could apply for funds for any or all components listed in the announcement through a competitive process. Box 1 lists cooperative agreement activities for public health genomics.

**Box 1. Program Activities Supported by Cooperative Agreements in Public Health Genomics Through the Centers for Disease Control and Prevention, 2003–2008**

1. Develop or expand leadership capacity in public health genomics.
2. Develop and implement population-based assessments and incorporate genomics into disease-specific data collection through surveillance and registries.
3. Implement or expand the use of genomics in program activities.
4. Educate the health care workforce, policy makers, and the public about the importance and role of family health history and genetic risk factors in disease etiology and prevention.
5. Prepare the chronic disease workforce for using genomic tools to reduce the burden of specific diseases, and teach them the benefits and limitations of genetic tests.

### State strategies

From 2003 through 2008, four states — Michigan, Minnesota, Oregon, and Utah — received cooperative agreement funds for public health genomics. Annual awards between $150,000 and $250,000 allowed each state to support 1 full-time staff member. Additional staff were hired part-time or as student interns; health departments also contributed staff time in kind (eg, for health education or web development support). States submitted annual progress reports to CDC.

Michigan, Oregon, and Utah chose to expand their existing state genetics plans, developed in 2002 with support from the Health Resources and Services Administration. They enhanced these plans with specific goals and activities that emphasized the role of genomics in chronic disease prevention programs (5–7). For example, new objectives in the Utah plan included educating health care providers and the public on genetic screening and testing; collecting and using family history in disease prevention; assessing the role of genetics in chronic disease clusters; developing a statewide family history database; and addressing state policy and ethical issues (8). Minnesota made a strategic decision to integrate genetic risk factors and strategies into state plans for preventing chronic diseases.

All 4 states integrated genomic information — particularly family health history — into prevention plans for 1 or more chronic diseases, including asthma, obesity and diabetes, heart disease, and stroke. The most detailed plans focused on cancer. Oregon’s comprehensive cancer control plan added an objective to increase awareness of cancer family history as a factor in individual cancer risk (9). Minnesota’s plan added objectives to promote appropriate referral and third-party payment for genetic risk assessment for cancer (10).

## Findings

Some key accomplishments are summarized in the following sections, corresponding with the categories in Box 1. References to published and online materials provide additional examples, details, and background information.

### Developing leadership capacity

Project staff in the 4 funded states identified “champions,” leaders within the health department who helped to develop partnerships and advisory groups. These groups varied in composition and scope, with members coming from various types of organizations: government agencies, academic and research institutions, health care plans, community groups, private businesses, genealogy organizations, and other entities. Oregon convened a broad Genetic Advisory Committee with clinical, public health, and academic members to gather input for Oregon’s Strategic Plan for Genetics and Public Health (6). Michigan formed the Michigan Cancer Genetics Alliance to promote translation of cancer genetics research into clinical and public health practice for cancer detection, prevention, and treatment (11). Utah’s Family Health History Taskforce provided leadership for public awareness, clinical applications, and methodology and research related to family health history (12). Minnesota developed a partnership with the Center for Public Health Education and Outreach at the University of Minnesota.

### Incorporating genomics into population-based assessments and surveillance

All 4 states had access to population-based data collected by state surveillance systems. Michigan, Oregon, and Utah added state-specific questions to the BRFSS (topics covered are listed in Table 1). Results of these surveys described the influence of family health history on respondents’ perceived risk, behaviors, health care experiences, and awareness of genetic tests marketed directly to the public (13–18). Utah used the Youth Risk Behavior Surveillance System (YRBSS) to assess high school students’ knowledge of genetics and found that black, Native American, and Pacific Islander students were less likely than others to report having received lessons on family health history (8, pp. 31–36). Oregon added questions on family health history to the Pregnancy Risk Assessment and Monitoring System (PRAMS) and the General Knowledge Survey (19). The Oregon Health Authority compiled an online summary of data from surveys conducted in the 4 pilot states and other state surveillance systems during 2001 through 2010 (20).

**Table 1:** Genomic Information Collected by the Behavioral Risk Factor Surveillance System (BRFSS), by State, 2003–2008^a^

Information Category	2003	2004			2005			2006			2007			2008		
	OR	MI	MN	OR	MI	OR	UT	MI	OR	UT	MI	OR	UT	MI	OR	UT
**Family history prevalence**																
Asthma	—	—	—	—	—	—	—	—	—	X	—	—	—	—	—	—
Breast or ovarian cancer	—	—	—	—	—	—	—	X	—	—	—	—	—	X	—	—
Cardiovascular disease	—	—	—	—	—	—	—	—	—	—	X	X	—	—	—	—
Colorectal cancer	—	—	—	—	X	—	—	—	—	—	—	—	—	—	X	—
Diabetes	X	—	X	X	—	X	—	—	X	—	—	—	X	—	—	X
Chronic disease (general)	—	—	—	—	—	—	X	—	—	X	—	—	X	—	—	X
**Awareness and collection of family health history**	—	X	—	—	X	—	X	—	—	X	—	X	X	—	—	X
**Awareness and use of DTC genetic testing**	—	—	—	—	—	—	—	X	—	X	—	X	X	—	X	X
**Opinions on the use of dried blood spots in research**	—	—	—	—	—	—	—	—	—	—	—	—	—	X	—	—

Abbreviations: OR, Oregon; MI, Michigan; MN, Minnesota; UT, Utah; —, data not collected; DTC, direct-to-consumer.

a State participation in BRFSS and survey questions varied by year.

The Michigan public health genomics program collaborated with the Michigan Cancer Surveillance Program (MCSP), the state’s cancer registry, to assess completeness of cancer family history in oncology patients’ charts. They found that more than 80% of 853 charts documented the presence or absence of a family history of cancer and the tumor site; however, fewer than 10% included age at cancer diagnosis, a key indicator for assessing potential genetic risk (21). On the basis of these findings, MCSP in 2007 began requiring that case reports include family history data, making it possible to ascertain cancer patients at high risk for familial cancer.

Michigan also developed a novel, population-based surveillance system for sudden cardiac death among the young (SCDY). Michigan used these data to bring attention to SCDY as a public health problem, identifying an underlying genetic cause in a large proportion of cases (22,23).

### Using genomic information in disease prevention programs

State programs used population-based data to inform educational activities, policies, and standards for using family health history to enhance early disease detection and prevention (Table 2). Michigan and Minnesota integrated family history risk assessments and educational materials into the screening process for WISEWOMAN programs (24). The success of these efforts helped to establish family history risk assessment as a service component of subsequent WISEWOMAN grants.

**Table 2:** Selected Highlights, Public Health Genomics Programs by State, 2003–2008

Topic Area	Michigan	Minnesota	Oregon	Utah
Disease-specific prevention plans and programs	Asthma Cancer CVD Obesity	Asthma Cancer CVD Diabetes Disabilities	Cancer CVD Diabetes Nutrition and physical fitness	Asthma Cancer CVD
Policies, guidelines, and standards	Cancer registry Electronic health record WISEWOMAN	WISEWOMAN	Privacy Health plan Birth defects registry	Medical family history
Education and training	Public health workers Secondary teachers Health professionals	Public health workers Graduate students Health professionals	Public health workers Health professionals	Public health workers Underserved communities Health professionals

Abbreviations: CVD, cardiovascular disease; WISEWOMAN, Well-Integrated Screening and Evaluation for Women Across the Nation.

Michigan integrated family history questions into the baseline participant survey of its Healthy Homes University program. This program is funded by the US Department of Housing and Urban Development (DHUD) to reduce asthma triggers in low- to moderate-income households that include a child with asthma. Sixty-five percent of children with diagnosed asthma in Michigan’s survey had at least 1 first-degree relative with asthma. By identifying affected relatives, Michigan’s program was able to offer education and products to reduce asthma triggers to 150 additional persons with asthma in 92 of the first 162 households enrolled in the project (25). This innovative approach was recognized by DHUD and has been sustained in Michigan (26). Utah also addressed asthma prevention, analyzing BRFSS data for asthma family history and conducting asthma genomics workshops in 2006 and 2007.

From 2006 through 2009, the Michigan Colorectal Cancer Screening Program offered colorectal cancer screening to asymptomatic low-income, uninsured, and underinsured persons in 3 counties with high colorectal cancer mortality rates. The program linked local health departments with genetics services and provided data for use in communicating with program participants. Of the approximately 1,500 adults screened, 177 were referred to a genetic counselor. In Minnesota, the Department of Health integrated family history educational materials into the Sage Screening Program for breast and cervical cancer among underserved women aged 40 or older (27).

The Oregon public health genomics program focused on policy development, supporting the legislatively established Advisory Committee on Genetic Privacy and Research and facilitating a new Genetics Advisory Committee that served the Oregon Health Services Commission and Oregon’s Medicaid program. In 2007, the commission approved all of the committee’s recommendations on coverage of genetic services under Oregon’s Medicaid program. These services included testing for the *BRCA1* or *BRCA2* mutation in patients without cancer who met US Task Force on Preventive Services guidelines as well as genetic testing for Lynch syndrome according to National Comprehensive Cancer Network guidelines. The Oregon public health genomics program analyzed state legislation, drafted testimony, and provided information on legislative issues, such as the development of an Oregon Birth Defects Registry, the disclosures permitted by the Oregon Genetic Privacy Act, and the use of genetic information in criminal cases (28).

### Educating the health workforce, policy makers, and the public

To increase awareness and understanding of the use of genomics — particularly family health history — in disease prevention, the states provided courses, seminars, and lectures and developed educational materials for public health practitioners, health care providers, policy makers, teachers, students, and the public (Table 2). To train state public health practitioners working in cancer control and prevention, Michigan produced a seminar series titled Cancer Genomics for Public Health. Oregon hosted a brown bag series on genomics for state public health workers, and Minnesota collaborated with the University of Minnesota’s School of Public Health to organize courses on genomics for the university’s annual Summer Public Health Institutes. Minnesota conferences on genomics in 2004 and 2005 attracted more than 200 participants.

Michigan developed continuing education modules on genomics, family history, and diabetes, which were accessed by more than 300 health care providers. Michigan also helped create a core lecture for first-year medical students at Wayne State University and collaborated with a regional health care network in southeastern Michigan to establish an annual grand rounds lecture on genomics for physicians and medical residents. Oregon explained how to use family history to identify patients at high risk for diabetes, colorectal cancer, breast cancer, and hyperlipidemia in *CD Summary*, a bi-weekly publication distributed to approximately 18,000 persons, including all licensed health care professionals in Oregon (29).

Michigan developed the Genetics to Genomics Workshop, which enrolled approximately 200 secondary school teachers in 2005 through 2006. Utah partnered with the University of Utah’s Genetic Science Learning Center to establish the Community Genetics Education Network, which provides genetic education to underserved and underrepresented minority communities. Utah developed and field tested the curriculum Using Family History to Improve Your Health to teach high school students about hereditary, lifestyle, and environmental components of chronic disease and to help them understand disease risk. The curriculum met national and state health education and biology standards and was made available in Spanish.

For health care providers and the public, Minnesota developed and broadly disseminated fact sheets on family history of chronic diseases. Utah developed the Family History Toolkit to help people collect and interpret their family health history (30). The English version of the tool kit was downloaded from the Utah website more than 75,000 times, the Spanish version nearly 20,000 times, and a version for seniors more than 11,000 times. All 4 states also promoted public awareness of family health history with media activities each November, when the US Surgeon General encourages Americans to discuss family health history as part of the Thanksgiving Day observance. For their efforts, Utah received the 2006 Silver Award for Excellence in Public Health Communication from the National Public Health Information Coalition.

## Effect of Early Pilot Efforts

Four state health departments developed pilot programs that integrated human genomics into core public health functions and programs. They were encouraged to explore local opportunities and build on existing resources, an approach that yielded novel projects that were difficult to evaluate as a whole. Nevertheless, they achieved important successes in 3 major areas: 1) conducting population-based assessments and surveillance to develop evidence for the use of genomic information and family history in state disease prevention efforts; 2) demonstrating the integration of this information into existing disease prevention programs, such as WISEWOMAN and Healthy Homes University; and 3) developing, disseminating, and documenting the uptake of educational materials for the health workforce, policy makers, and the public.

The pilot programs also delivered some useful lessons on strategies and challenges for integrating genomics into public health programs for chronic disease prevention (Box 2) (31). Perhaps most important, they showed that there is no single model for success; rather, approaches must be adapted to state culture, infrastructure, and resources. The support of health department leadership, when available, proved to be crucial. Opportunities to promote the development of genomics activities within other state and local health department programs helped leverage resources and integrate genomics into practice. External partnerships were also important; in addition to other state agencies and universities, these sometimes included nontraditional partners such as genealogy groups, businesses, and news media. Features that helped sustain some activities beyond the end of the cooperative agreement period included relevance to existing programs, need for no more than incremental changes, visibility, and demonstrated effect. Key challenges included the scarcity of actionable evidence, competing priorities, scarce financial and personnel resources, and communication barriers between public health and genetics professionals.

**Box 2. Integrating Genomics into Public Health Chronic Disease Prevention Programs: Successful Strategies and Challenges**

**Successful strategies**

- Obtain support of health department leadership and decision makers.
- Seek partnership with early adopters of genomics among disease prevention programs.
- Meet programs at their level of interest in a way that is flexible, responsive, and open to ideas.
- Engage local public health departments; they are an important setting for integrating genomic information into practice.
- Develop external partnerships; consider nontraditional partners, such as genealogists, private businesses, community members, information technology experts, pharmaceutical companies, and the news media.
- Develop communications and educational materials that are easy to understand, practical, relevant, and accessible to their intended audiences.
- Focus on activities that are relevant to existing programs, require only incremental changes, demonstrate value and impact, and are likely to be sustained.

**Challenges**

- Evidence-based guidelines and professional standards for integrating family health history, genetic testing, and genetic risk factors into disease prevention programs are limited.
- Disease prevention programs have many competing priorities and are reluctant to integrate genomics into their activities without a mandate from their funding source.
- Minimal financial support for public health genomics is available from state health departments and federal agencies.
- Cooperative agreements that exclude research and clinical services limit the ability of health departments to advance the field of public health genomics.
- Funds are limited or lacking to cover clinical genetic services and testing for persons who are uninsured, underinsured, or insured through Medicaid.
- No model or best practices exist for the organizational structure of state public health genomics programs.
- Most health departments have few staff members with training in genetics.
- Lack of a common vocabulary and other barriers hinder communication between public health practitioners and genetics experts.

Although the pilot programs did not set out to measure health outcomes, they created a foundation for successes in subsequent years. Michigan and Oregon sustained their public health genomics programs with limited state funding and in 2008 received new, 3-year cooperative agreements for implementing evidence-based recommendations on hereditary cancer through education, surveillance, and policy development. The new programs built on infrastructure developed during the pilot programs to carry out targeted interventions. Michigan used cancer registry data from 2006 through 2007 to identify more than 15,000 people needing evaluation for *BRCA1* or *BRCA2* or Lynch syndrome by the end of 2012. The Michigan Public Health Genomics Program and the MCSP established a bidirectional reporting system, which returns information on persons with characteristics suggesting hereditary cancer to the 150 health care institutions that report to the MCSP (32). In addition, the state partnered with payers to extend insurance coverage for these services to over 7 million Michigan residents (33).

The experiences of Michigan, Minnesota, Oregon, and Utah are useful to a growing number of states — including Connecticut, Ohio, and Georgia — that are developing their own approaches (34–36). Expectations for genomic medicine have moderated considerably during the last decade, but evidence-based recommendations now support the use of several genomic health applications to prevent chronic diseases (37). In particular, nearly 2 million Americans are at increased risk for early-onset cancer or heart disease because they have a hereditary breast or ovarian cancer mutation (*BRCA1* or *BRCA2*), Lynch syndrome, or familial hypercholesterolemia; most people with these conditions are not aware they have them, yet early detection and intervention could save their lives (38).

CDC’s Office of Public Health Genomics website offers materials for implementing interventions that proceed from evidence-based recommendations (39). Many of these are products of the pilot programs described in this article. They show how to build partnerships between public health and private health care, enhance cancer registry reporting for selected hereditary cancers, develop coverage for recommended clinical services, devise surveillance indicators for tracking health impact, and deliver information to health care providers, policy makers, and the public.
